# The Effect of Hydroxychloroquine on Symptoms of Knee Osteoarthritis: A Double-Blind Randomized Controlled Clinical Trial

**Published:** 2013-09

**Authors:** Mohammadhassan Jokar, Zahra Mirfeizi, Kamran Keyvanpajouh

**Affiliations:** 1 Department of Internal Medicine, Imam Reza Hospital, Mashhad University of Medical Sciences, Mashhad, Iran;; 2 Department of Rheumatology, Imam Khomeini Hospital, Urmia University of Medical Sciences, Urmia, Iran

**Keywords:** Osteoarthritis, Knee, Hydroxychloroquine

## Abstract

**Background: **Osteoarthritis is a degenerative joint disorder of articular cartilage and is the most common type of arthritis in the elderly. There are only a few reports regarding the use of Hydroxychloroquine in the treatment of osteoarthritis.

**Methods: **To investigate the effects of Hydroxychloroquine on the symptoms of mild to moderate knee osteoarthritis (Kellgren and Lawrence grade II and III), we performed a double-blind, placebo-controlled study in 44 patients. The patients were randomly assigned to two groups: one group received Hydroxychloroquine pills (200 mg twice daily) and the other group received placebo pills. Symptoms were assessed by the Western Ontario and McMaster Universities Osteoarthritis Index (WOMAC) at baseline and at the end of weeks 4, 8, 12, 16, 20, and 24.

**Results: **Approximately, 98% of the patients were women at an average age of 47 years. There was no significant difference in the baseline characteristics between the two groups. In the placebo group, maximum improvement occurred at the 4^th^ week; and during the remaining time, there was no significant improvement. In the Hydroxychloroquine group, maximum improvement occurred at the 8^th^ week and persisted over the entire remaining follow-up period. There were significant differences between the two groups regarding the degree of reduction in the WOMAC total score and the WOMAC subscales scores of pain, stiffness, and function at the end of weeks 4, 8, 12, 16, 20, and 24.

**Conclusion:** Hydroxychloroquine conferred significant improvement in the symptoms of mild to moderate knee osteoarthritis in our patients and may, accordingly, be recommended for knee osteoarthritis treatment.

## Introduction

Osteoarthritis is the most common type of arthritis.^1^ Its high prevalence, especially in the elderly, and the high rate of disability related to the disease make it a leading cause of disability in the elderly.^2^ Because of the aging of world populations and the increasing prevalence of obesity as a major risk factor, the occurrence of osteoarthritis is on the rise.^3^

Treatment of osteoarthritis can be frustrating for patients and physicians.^4^ The goals of the management of patients with osteoarthritis are to control pain and swelling, minimize disability, and improve the quality of life. Currently, the pharmacological treatment of osteoarthritis is primarily aimed at controlling symptoms and analgesics and non-steroidal anti-inflammatory drugs (NSAIDs) are commonly prescribed. There are at present no specific pharmacologic therapies that can slow the progression of this condition.^2^

Antimalarial agents have immunomodulatory and anti-inflammatory properties, although their precise mechanism of action in rheumatic diseases is unknown. The anti-inflammatory properties of the antimalarials include effects on the arachidonic acid cascade, by downregulation of phospholipase A2 and C, which contribute to the production of proinflammatory prostaglandins and lipid peroxidation.^5^^,^^6^ Lipid peroxidation is thought to play a role in apoptosis. Over the last two decades, there has been increasing evidence showing the importance of classic apoptosis in the creation of osteoarthritis.^7^ Antimalarial agents also have antioxidant properties and may provide protection against tissue damage by free radicals.^5^^,^^6^

The purpose of the present study was to investigate the potential effect of Hydroxychloroquine (HCQ) on the symptoms of knee osteoarthritis.

## Patients and Methods

This 24-week, randomized, double-blind, parallel-group study was conducted on knee osteoarthritis patients. All the patients fulfilled the American College of Rheumatology classification criteria for knee osteoarthritis.^8^ Eligible patients were those who met all of the following criteria: 1) primary knee osteoarthritis; 2) knee osteoarthritis Kellgren and Lawrence grade II or III;^9^ 3) knee pain for at least the preceding 6 months; 4) minimum age of 30 years; and 5) literacy. Patients were excluded if they had any of the following: 1) secondary osteoarthritis; 2) knee arthroscopy during the preceding 6 months; 3) intra-articular injection of corticosteroids during the last 6 months; 4) presence of other inflammatory diseases; 5) history of hypersensitivity to antimalarial drugs; and 6) any kind of eye disease.

The trial was registered in the Iranian Registry of Clinical Trials database, accessible at www.rct.ir (IRCT138709121479N1). The study protocol received approval from the Ethics Committee of Mashhad University of Medical Sciences, and all the patients provided written informed consent prior to study participation.

Patients were enrolled from outpatient clinics. All the patients underwent a baseline eye examination by an ophthalmologist. The patients were thereafter divided into two groups via a random number table: the first group received 200 mg HCQ (Hydroxychloroquine; Amin Pharmaceutical Co., Tehran, Iran) with dosage of 200 mg twice daily for 6 months and the second group received placebo tablets with the same schedule. The HCQ and placebo tablets were identical in shape and appearance. The placebo pills were produced by the Faculty of Pharmacy, Mashhad University of Medical Sciences. All the drugs were labeled with the randomization numbers of the participants. The patients and the staff members involved in data collection were unaware of the group assignment. 

The patients were allowed to use their usual painkillers (analgesics and NSAIDs). However, they were told to discontinue their analgesics and NSAIDs 48 hours before the physician’s visit to avoid confounding the test results. The patients were also asked to record the name and number of the analgesics and NSAIDs that they consumed during each day. All the patients were requested at each visit to report any eventual side effect. 

All the patients were trained how to answer the Western Ontario and McMaster Universities Osteoarthritis Index (WOMAC).The WOMAC index questionnaires were completed by each patient at the baseline visit and at the end of weeks 4, 8, 12, 16, 20, and 24. The WOMAC measures five items for pain (score range 0–50), two for stiffness (score range 0–20), and 17 for functional limitation (score range 0–170). 

The data were analyzed by SPSS statistical software (version 16) (SPSS Institute, USA). The numerical variables are described by mean and standard deviation. The baseline characteristics were compared between the groups using the Fisher exact text for the qualitative variables and Student’s *t* test or the Mann–Whitney U test for the quantitative variables (chosen according to the nature or distribution of these variables). A repeated measure ANOVA was performed to assess the change over time in the WOMAC total score and the WOMAC subscales scores of pain, stiffness, and function. The level of significance was a P value smaller than 0.05.

## Results

**Figure 1:**
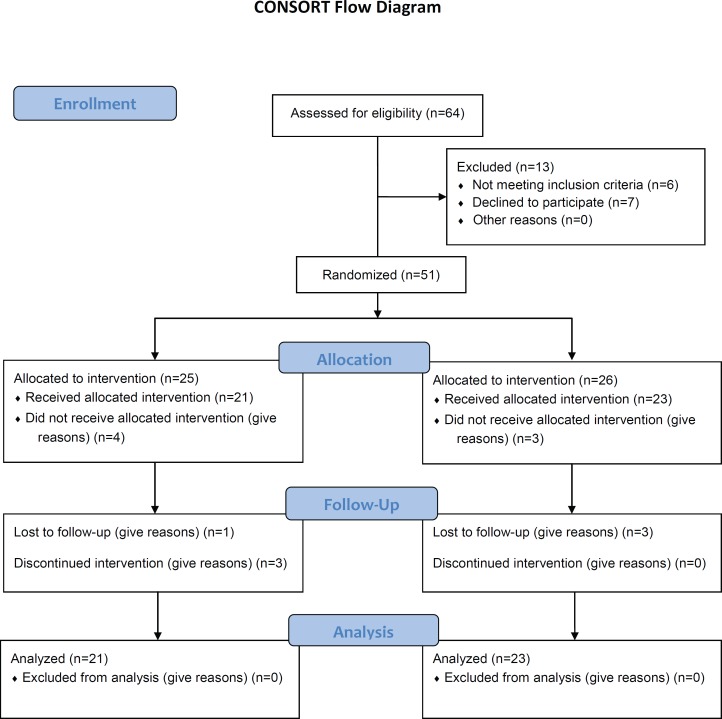
This flowchart depicts the process of patient assignment to the trial arms. F/U: Follow-up

Sixty-four patients were introduced to the study from our Rheumatology Clinics. Of them, six patients were not eligible and seven refused to give consent. Fifty-one patients were, therefore, randomized into two groups (placebo group=26, HCQ group=25); however, forty-four patients completed the study (placebo group=23, HCQ=21): four patients were lost to follow-up (placebo group=3, HCQ group=1) and 3 patients (placebo group=0, HCQ group=3) discontinued the treatment due to drug side effects (figure 1). Both groups were well-matched for baseline and demographic characteristics (table 1). 

**Table 1:** Baseline characteristics of the studied patients

**Variable**	**Placebo group (No: 23)** **(mean±SD)**	**HCQ group (No: 21)** **(mean±SD)**	**P value**
Age (y)	47.60±8.54	48.28±11.14	0.823
Female sex, number (%)	22 (95)	21 (100)	0.167
BMI	25.39±2.79	25.57±3.05	0.840
Kellgren & Lawrence			0.763
Grade II	12	11	
Grade III	11	10	
WOMAC total score	119.70±24.78	124.71±44.71	0.644
WOMAC pain score	26.74±8.00	30.62±8.66	0.130
WOMAC stiffness score	8.78±6.18	6.57±4.69	0.192
WOMAC function score	84.17±22.63	87.52±36.36	0.719

BMI: Body Mass Index; WOMAC: Western Ontario and McMaster Universities Osteoarthritis Index

**Figure 2:**
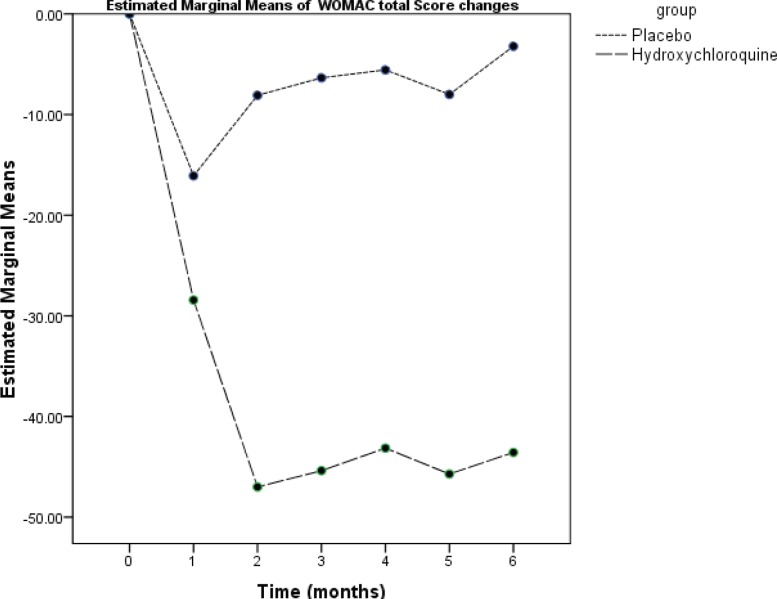
This graph illustrates the mean changes from baseline in the Western Ontario and McMaster Universities Osteoarthritis Index (WOMAC) total score over 24 weeks of treatment in the studied patients

**Figure 3:**
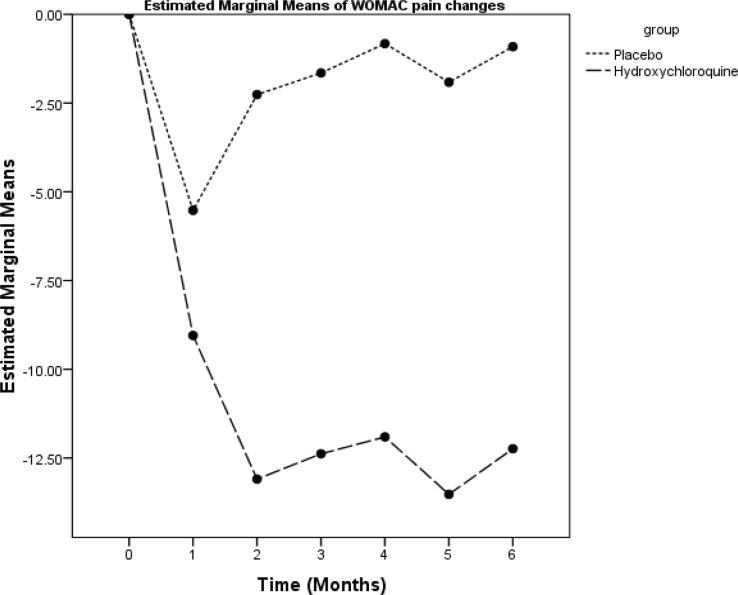
This graph illustrates the mean changes from baseline in the Western Ontario and McMaster Universities Osteoarthritis Index (WOMAC) pain score over 24 weeks of treatment in the studied patients

**Figure 4:**
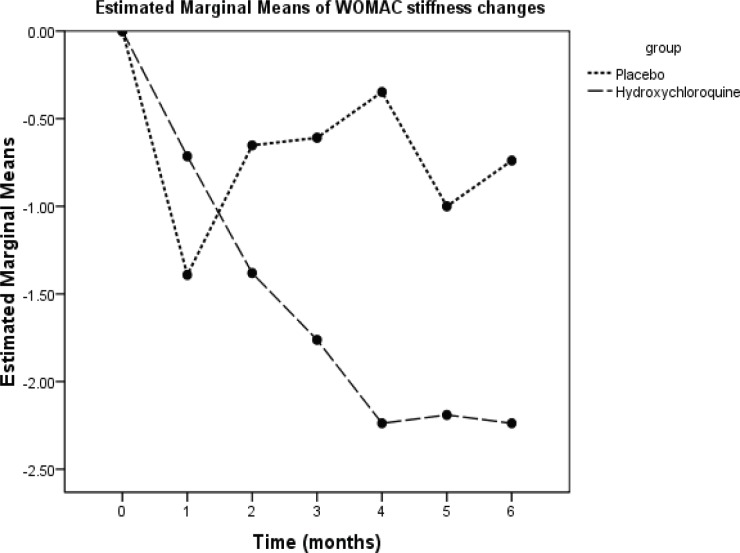
This is an illustration of the mean changes from baseline in the Western Ontario and McMaster Universities Osteoarthritis Index (WOMAC) stiffness score over 24 weeks of treatment in the study population

**Figure 5:**
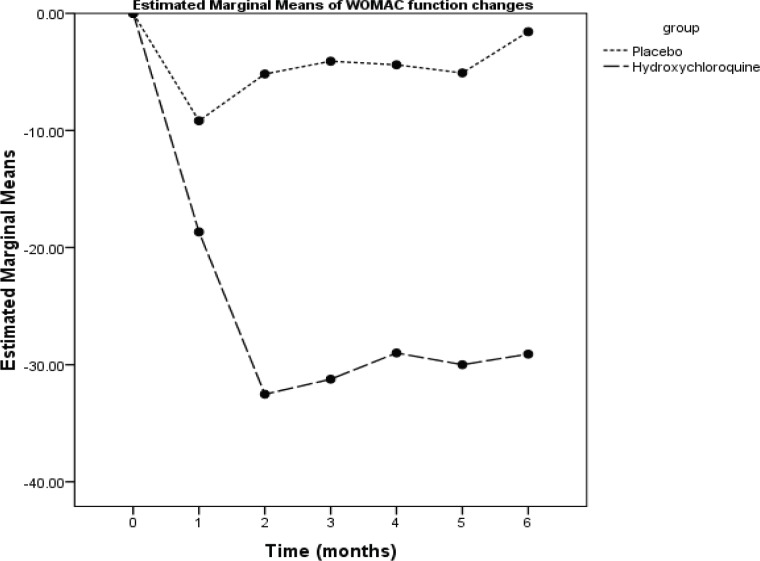
This is a represntation of the mean changes from baseline in the Western Ontario and McMaster Universities Osteoarthritis Index (WOMAC) function score over 24 weeks of treatment in the study population

The WOMAC scores of the subjects in the two groups at various time points of the study are summarized in table 2. A significant interaction effect was observed between time and the group (repeated measure ANOVA) in the WOMAC total score and the WOMAC subscales scores of pain, stiffness, and function. In the placebo group, maximum improvement occurred at the 4^th^ week and there was no significant improvement during the remaining time. In the HCQ group, maximum improvement occurred at the 8^th^ week and lasted over the entire remaining follow-up period. There were significant differences between the two groups as regards the degree of reduction in the WOMAC total score and the WOMAC subscales scores of pain, stiffness, and function at weeks 4, 8, 12, 16, 20, and 24 (figures 2-5).

**Table 2:** Mean WOMAC scores over time in the studied patients

**Parameters**	**Baseline**	**Day 30**	**Day 60 **	**Day 90**	**Day 120**	**Day 150**	**Day 180**	**F**	**P value**
WOMAC pain								4.12	0.049
Placebo	26.74±8.00	21.22±10.13	24.48±8.51	25.09±11.15	25.91±10.61	24.83±8.31	25.83±10.07		
HCQ	30.62±8.66	21.57±7.78	17.52±9.13	18.24±10.94	18.71±10.24	17.10±8.16	18.38±9.72		
WOMAC Stiffness								8.63	0.005
Placebo	8.78±6.18	7.39±3.82	8.13±3.30	8.17±4.21	8.43±4.49	7.78±4.14	8.04±4.237		
HCQ	6.57±4.69	5.86±5.32	5.19±5.08	4.81±5.39	4.33±5.51	4.38±5.40	4.33±4.004		
WOMAC Function								4.91	0.032
Placebo	84.17±22.63	75.00±24.89	79.00±24.30	80.09±31.16	79.78±33.29	79.09±30.03	82.61±26.26		
HCQ	87.52±36.36	68.86±37.54	55.00±30.31	56.29±33.19	58.52±33.29	68.80±34.98	58.43±35.44		
WOMAC Total								5.74	0.021
Placebo	119.70±24.78	103.61±34.32	111.61±30.99	113.35±37.90	114.13±39.92	111.70±38.20	116.48±37.43		
HCQ	124.71±44.71	96.28±44.91	77.71±41.66	79.33±46.88	81.57±45.86	79.00±45.52	81.14±46.53		

WOMAC: Western Ontario and McMaster Universities Osteoarthritis Index

There was a statistically significant difference between the two groups vis-à-vis the average number of painkiller pills consumed during the trial (0.74 pill per day in the HCQ group and 0.96 pill in the placebo group; P=0.035). Three patients in the HCQ group discontinued the treatment due to drug side effects (skin rash in 2 and vertigo in one), whereas there were no drug side effects in the placebo group. The difference between the two groups with respect to the frequency of drug side effects was statistically significant (P=0.001).

## Discussion

The knee joint is commonly afflicted by osteoarthritis. As much as knee osteoarthritis is a disease of high prevalence in world populations and is associated with high morbidity, the treatment has shown little progress in the last decades.^10^^,^^11^ Pharmacologic treatment categories for osteoarthritis are typically set up to designate whether drugs are symptom-relieving or disease-modifying. Nonetheless, the evidence has thus far proved insufficient as to which drugs have optimal disease-modifying properties in osteoarthritis.^2^

Antimalarial agents have immunomodulatory and anti-inflammatory properties, and the effectiveness of these drugs in the therapy of some rheumatic diseases has been well known in the medical literature for many years.^5^^,^^6^ Be that as it may, their precise mechanism of action in rheumatic diseases is still unknown.

There are only a few reports regarding the use of HCQ in osteoarthritis. Indeed, there is only one report on the use of HCQ in knee osteoarthritis. Herval de Lacerda Bonfante et al.^12^ in a controlled, randomized, double-blind study assessed the effectiveness of HCQ on knee osteoarthritis and concluded that although their two groups of HCQ and placebo exhibited improvement, HCQ had no superiority over the placebo in the treatment of knee osteoarthritis. Bryant, Desrosier, and Carpenter^13^ treated 8 patients with erosive hand osteoarthritis and obtained satisfactory results in 6 patients, all of whom showed improvement in a period ranging from 7 weeks to 7 months. Gianantonio Saviola et al.^14^ compared the efficacy of Clodronate (a bisphosphonate) with HCQ for treating active erosive osteoarthritis of the hand and demonstrated that while the former probably played an efficacious role in the treatment of active erosive osteoarthritis, the latter seemed to be ineffective. Vuolteenaho et al.^15^ found that HCQ suppressed nitric oxide production induced by IL-1 β in osteoarthritic cartilage and concluded that this drug could be useful in treating this disease. 

The present study was performed to investigate the effect of HCQ on mild to moderate knee osteoarthritis symptoms. The average age of our patients was less than that in the Herval de Lacerda Bonfante^12^ study (48 years vs. 60 years). The lower age of our patients may be due to the affliction of younger people by knee osteoarthritis in Iran compared with some other countries.^16^^,^^17^ In contrast to the Herval de Lacerda Bonfante study, the results of our study showed statistically significant improvement in the total WOMAC score, WOMAC pain score, WOMAC stiffness score, and WOMAC function score in the patients using HCQ. Although drug side effects statistically were more frequent in the HCQ group, the symptoms were mild to moderate and were improved immediately after the discontinuation of HCQ. 

The results of the present study showed that HCQ improved the symptoms of knee osteoarthritis in our patients. Nevertheless, no conclusion can be drawn apropos the disease-modifying action of HCQ because the focus of the study was merely on clinical changes. Larger new clinical trials are required to evaluate not only the symptoms but also the joint space if one is to demonstrate the validity of the use of HCQ as a disease-modifying drug. 

## Conclusion

HCQ conferred significant improvement in the symptoms of mild to moderate knee osteoarthritis in our patients and can, therefore, be deemed useful in the treatment of knee osteoarthritis. 
